# Enalapril Is Superior to Lisinopril in Improving Endothelial Function without a Difference in Blood–Pressure–Lowering Effects in Newly Diagnosed Hypertensives

**DOI:** 10.3390/biomedicines11123323

**Published:** 2023-12-15

**Authors:** Attila Nagy, Réka Májer, Judit Boczán, Sándor Sipka, Attila Szabó, Enikő Edit Enyedi, Ottó Tatai, Miklós Fagyas, Zoltán Papp, László Csiba, Attila Tóth

**Affiliations:** 1 Department of Health Informatics, Institute of Health Sciences, Faculty of Health Sciences, University of Debrecen, 4032 Debrecen, Hungary; attilanagy@med.unideb.hu; 2 Department of Neurology, Faculty of Medicine, University of Debrecen, 4032 Debrecen, Hungary; majer.reka@med.unideb.hu (R.M.); boczan@med.unideb.hu (J.B.); csiba@med.unideb.hu (L.C.); 3 MTA–DE Cerebrovascular and Neurodegenerative Research Group, 4032 Debrecen, Hungary; 4 Division of Cardiology, Department of Cardiology, Faculty of Medicine, University of Debrecen, 4032 Debrecen, Hungaryfagyasmiklos@med.unideb.hu (M.F.); 5 Division of Clinical Physiology, Department of Cardiology, Faculty of Medicine, University of Debrecen, 4032 Debrecen, Hungary; szabo.attila@med.unideb.hu (A.S.); enyedi.eniko@med.unideb.hu (E.E.E.); tataiotto2003@gmail.com (O.T.); pappz@med.unideb.hu (Z.P.)

**Keywords:** Angiotensin–converting enzyme (ACE), ACE inhibitor, lisinopril, enalapril, clinical study, endothelial function, hypertension, carotis IMT, FMD

## Abstract

Angiotensin–converting enzyme (ACE) inhibitors are the primarily chosen drugs to treat various cardiovascular diseases, such as hypertension. Although the most recent guidelines do not differentiate among the various ACE inhibitory drugs, there are substantial pharmacological differences. Goal: Here, we tested if lipophilicity affects the efficacy of ACE inhibitory drugs when used as the first therapy in newly identified hypertensives in a prospective study. Methods: We tested the differences in the cardiovascular efficacy of the hydrophilic lisinopril (8.3 ± 3.0 mg/day) and the lipophilic enalapril (5.5 ± 2.3 mg/day) (*n* = 59 patients). The cardiovascular parameters were determined using sonography (flow-mediated dilation (FMD) in the brachial artery, intima-media thickness of the carotid artery), 24 h ambulatory blood pressure monitoring (peripheral arterial blood pressure), and arteriography (aortic blood pressure, augmentation index, and pulse wave velocity) before and after the initiation of ACE inhibitor therapy. Results: Both enalapril and lisinopril decreased blood pressure. However, lisinopril failed to improve arterial endothelial function (lack of effects on FMD) when compared to enalapril. Enalapril-mediated improved arterial endothelial function (FMD) positively correlated with its blood–pressure–lowering effect. In contrast, there was no correlation between the decrease in systolic blood pressure and FMD in the case of lisinopril treatment. Conclusion: The blood–pressure–lowering effects of ACE inhibitor drugs are independent of their lipophilicity. In contrast, the effects of ACE inhibition on arterial endothelial function are associated with lipophilicity: the hydrophilic lisinopril was unable to improve, while the lipophilic enalapril significantly improved endothelial function. Moreover, the effects on blood pressure and endothelial function did not correlate in lisinopril-treated patients, suggesting divergent mechanisms in the regulation of blood pressure and endothelial function upon ACE inhibitory treatment.

## 1. Introduction

Angiotensin–converting enzyme inhibitors (ACEi) are the primarily chosen drugs to treat hypertension [1,2] ⁠and heart failure [3,4]. There are ten approved ACEi drugs, which are generally considered to have similar medical efficacies [5]. In particular, two widely used drugs, lisinopril and enalapril, had similarly positive effects on heart failure mortality [6] or hospitalization [7]. A uniform class effect was also noted in patients with myocardial infarction [8], congestive heart failure [9], and hypertension [10].

It is important to consider that circulating ACE seems to be completely inhibited by endogenous inhibitors [11,12] such as serum albumin [13], suggesting that only tissue-bound enzymes can be modulated by ACE inhibitory drugs [13]. This later was supported by the observation that physiological serum albumin completely inhibited human serum ACE, while only partial inhibition was observed in isolated human blood vessels [13]. These findings pinpoint vascular endothelium as the site of action for ACEi drugs. Therefore, the apparent class effect for ACEi drugs is rather surprising in light of marked differences in their pharmacokinetics [5], lipophilicities [14], and elimination pathways [14,15]. Here, we tested two drugs, originally developed by Merck [16]. Enalapril and its lysine analog have a similar efficacy in a wide range of clinical studies [17,18,19], albeit lisinopril was co-developed by Merck and Zeneca in the clinical phase. Both drugs are approved for the treatment of hypertension and congestive heart failure.

Here we performed a prospective clinical study to investigate the effects of two ACEi drugs with different lipophilicities. Lisinopril represented hydrophilic, low-protein binding ACEi [20], which was contrasted by enalapril, a lipophilic, relatively high-protein binding drug of the same class [14,21]. ACEi drugs were initiated at the first diagnosis of hypertension and the biochemical efficacy of the treatment was confirmed. The effects of lisinopril and enalapril were tested on blood pressure (24 h ambulatory blood pressure monitoring), arterial stiffness (arteriography), and endothelial function (flow-mediated dilation, brachial artery) before initiation and after the administration (at least 30 days) of the ACEi drugs.

## 2. Materials and Methods

### 2.1. Patients

Our research took place at the Department of Neurology, Clinical Center of the University of Debrecen. Based on the study protocol, we included patients presenting at their primary care provider with newly diagnosed primary hypertension (ICD code I10H0)—GPs and occupational health physicians aided the patient enrollment process. Asymptomatic and untreated patients whose hypertension was confirmed by ABPM and who had not yet received antihypertensive treatment were included. All subjects were asymptomatic, predominantly middle-aged (active age), as identified by screening. Following the ABPM, a CT scan was also performed to detect asymptomatic abnormalities (e.g., silent brain infarction).

The exclusion criteria included extreme obesity (body mass index—BMI greater than 35 kb/m^2^), previous stroke, TIA, a poor general condition, a life expectancy of fewer than 5 years, and co-morbidities that may significantly affect the hypertensive patients: diabetes, severe heart disease, psychiatric disorders (including alcohol dependence), dementia, Parkinson’s disease, neuromuscular disorders, autonomic nervous system syndromes, inflammatory diseases, stroke, and TIA. A “silent” infarction or other organic abnormalities detected on cranial CT also resulted in exclusion. Pregnant or post-partum candidates were also not recruited for the investigation.

### 2.2. Methods

Medical history, demographic variables, the results of a physical investigation, and laboratory tests (serum electrolytes, renal function, blood glucose level, HbA1C, lipid profile, complete blood count, CRP, fibrinogen level, and urine analysis) were recorded at the initiation of the treatment.

These assessments were followed by 24 h ambulatory blood pressure monitoring (ABPM). Blood pressure was measured every 15 min during the daytime (from 6:00 to 22:00) and every 30 min at night (from 22:00 to 6:00). Based on the data, we determined the daytime and nighttime mean systolic and diastolic pressures and systolic and diastolic hyperbaric index. For the ABMP measurements, Cardiospy ABPM equipment from Labtech Ltd. (Debrecen, Hungary, Model: EC-ABP) was used.

A flow-mediated dilatation (FMD) measurement of the brachial artery was performed using a HP Sonos 5500 ultrasound device with a 10 MHz linear test transducer (National Utrasound, Tampa, FL, USA). A B-mode longitudinal section was obtained from the brachial artery above the antecubital fossa. A forearm cuff was inflated to 10–40 mmHg above the patient’s systolic pressure for 5 min. Upon the cuff release, the induced hyperemia promoted an increase in the shear stress-mediated NO release and subsequent vasodilatation. The FMD was expressed as the percentage increase in the resting diameter of the artery after the cuff release with the baseline arterial diameter as a reference.

Arterial stiffness measurements were performed using TensioClinic arteriography (TensioMed Ltd., Budapest, Hungary) [22]. This technique is based on the fact that the contraction of the heart initiates pulse waves in the aorta. The first wave becomes reflected from the aortic wall at the bifurcation, therefore a second reflected wave appears as a late systolic peak. The cuff detects both waves. The morphology of this second reflected wave depends on the stiffness of the large artery, the reflection time at 35 mmHg supra systolic pressure of the brachial artery, and the peripheral resistance-dependent amplitude. Arterial stiffness was assessed by determining the Augmentation Index (AIx) and the Pulse Wave Velocity (PWV). The AIx was calculated from the amplitudes of the first and second waves and represents the pressure difference between the late systolic peak and the early systolic peak divided by the pulse pressure. The PWV is the ratio of the jugular fossa-symphysis distance (which is anatomically identical to the distance between the aortic trunk and the bifurcation) to the reflection time at 35 mmHg supra systolic pressure on the brachial artery. Brachial artery FMD is a technique for estimating the endothelial function in large arteries [23].

The data were evaluated using pairwise and correlation analyses. Assuming a normal distribution was generally avoided in the statistical analysis. One reason for this is that some sets of data did not show a normal distribution. The second is that we wanted to avoid false positive correlations caused by outliers in the datasets with relatively small observation numbers. The Kruskal–Wallis test was used for parameters from two populations (such as those treated with enalapril or lisinopril), while the Wilcoxon test was performed when the parameters before and after (paired) were evaluated. Spearman’s correlation analysis was performed when correlations were addressed.

## 3. Results

Individuals with primary hypertension were recruited immediately after their diagnosis. Two groups were formed according to the initiated medical therapy. One group was treated with lisinopril (*n* = 31), while the other was treated with enalapril (*n* = 28). The general clinical parameters are summarized in Table 1. Unfortunately, many patients did not volunteer for the multiple-day follow-up study.

**Figure 1:**
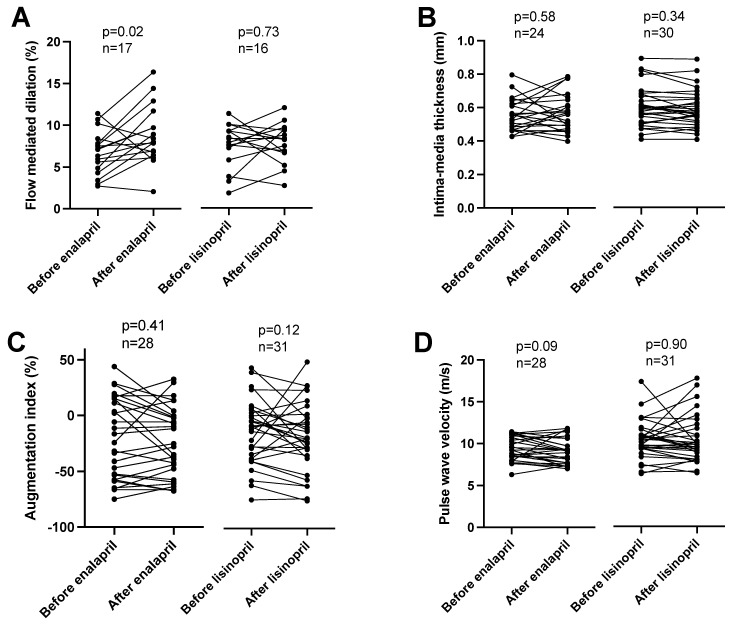
Different effects of enalapril and lisinopril on arterial endothelial function in hypertensive patients. Patients were tested for hypertension in the outpatient facility of the Department of Neurology. Newly identified hypertensive patients were enrolled in the study. Vascular parameters, such as flow-mediated dilation (panel (**A**)), intima-media thickness (panel (**B**)), augmentation index (panel (**C**)), and pulse wave velocity (panel (**D**)) were determined. Each symbol represents an individual patient. Lines connect values before and after enalapril or lisinopril treatment. Significant differences between values determined before and after the initiation of ACEi medication are labeled by the *p* values. Statistical differences were calculated by the Wilcoxon test.

The effects of the ACEi medications were tested by measuring the flow-mediated dilation (FMD) in the brachial artery after prolonged cuff use. The treatment of patients with enalapril improved the endothelial-mediated dilation (an increase of FMD from 6.7 ± 0.6 to 8.8 ± 0.8%, mean ± SEM, *n* = 17, Figure 1A), while the treatment with lisinopril was without effects (FMD before 7.5 ± 0.7 vs. after 7.7 ± 0.6% lisinopril treatment, mean ± SEM, *n* = 16, Figure 1A). The intima-media thickness of the carotid artery was unaffected by both enalapril (intima-media thickness before 0.55 ± 0.02 vs. after 0.57 ± 0.02 mm, mean ± SEM, *n* = 24, Figure 1B) and lisinopril (intima-media thickness before 0.60 ± 0.02 vs. after 0.59 ± 0.02 mm, mean ± SEM, *n* = 30, Figure 1B). Vascular stiffness was also tested with functional measurements. No significant effects were noted on the augmentation index (enalapril before: 20.1 ± 6.8, after: 23.9 ± 5.9, mean ± SEM, *n* = 28; lisinopril before: 8.2 ± 5.5, after: 12.8 ± 5.8, mean ± SEM, *n* = 31; Figure 1C) or pulse wave velocity determined using arteriography (enalapril before: 9.4 ± 0.3, after: 9.1 ± 0.3, mean ± SEM, *n* = 28; lisinopril before: 10.3 ± 0.4, after: 10.4 ± 0.5, mean ± SEM, *n* = 31; Figure 1C).

**Figure 2:**
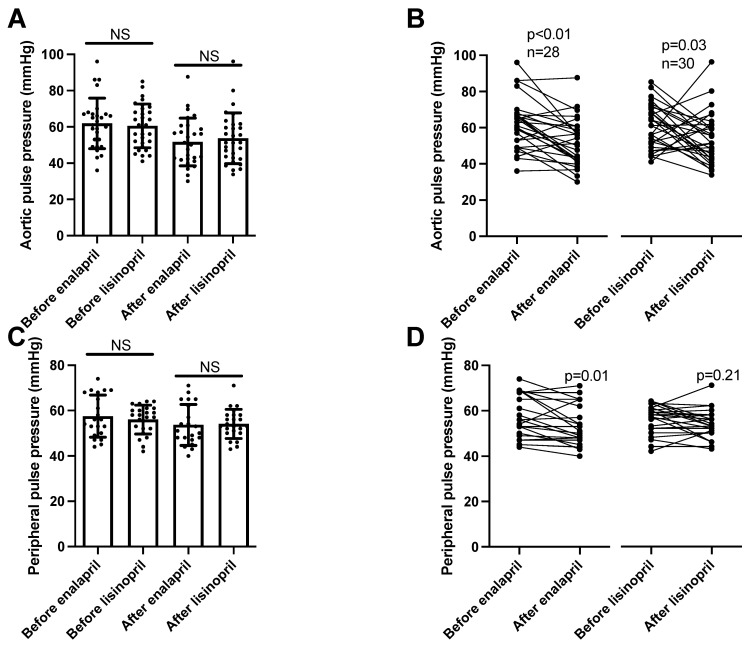
Similar effects of enalapril and lisinopril on pulse pressure in hypertensive patients. Newly identified hypertensive patients were enrolled in the study at the outpatient facility of the Department of Neurology. Systolic and diastolic blood pressure values were determined using 24 h ambulatory blood pressure monitoring (peripheral blood pressure) and arteriography (aortic blood pressure). The difference between the systolic and diastolic blood pressure values was calculated to yield pulse pressure. Each symbol represents an individual patient. Bars represent the mean and SD for aortic (panel (**A**)) and peripheral (panel (**C**)) pulse pressure values. No significant differences were found among the groups (indicated by NS) by Kruskal–Wallis tests. Lines connect values before and after enalapril or lisinopril treatment in the case of aortic (panel (**B**)) and peripheral (panel (**D**)) pulse pressure values. Significant differences between values determined before and after the initiation of ACEi medication are labeled by the *p* values. Statistical differences were calculated by the Wilcoxon test.

Aortic pulse pressure was similarly affected by both ACE inhibitors (enalapril before: 62 ± 3, after: 52 ± 2 mmHg, mean ± SEM, *n* = 28; lisinopril before: 61 ± 2, after: 54 ± 3 mmHg, mean ± SEM, *n* = 30; Figure 2A), being lowered by both lisinopril and enalapril (Figure 2B). Peripheral pulse pressure values were also similar among the groups (enalapril before: 58 ± 2, after: 54 ± 2 mmHg, mean ± SEM, *n* = 21; lisinopril before: 56 ± 1, after: 54 ± 1 mmHg, mean ± SEM, *n* = 23; Figure 2C). However, decreases at the level of the individual patients were only significant in the enalapril-treated patients (Figure 2D).

**Figure 3:**
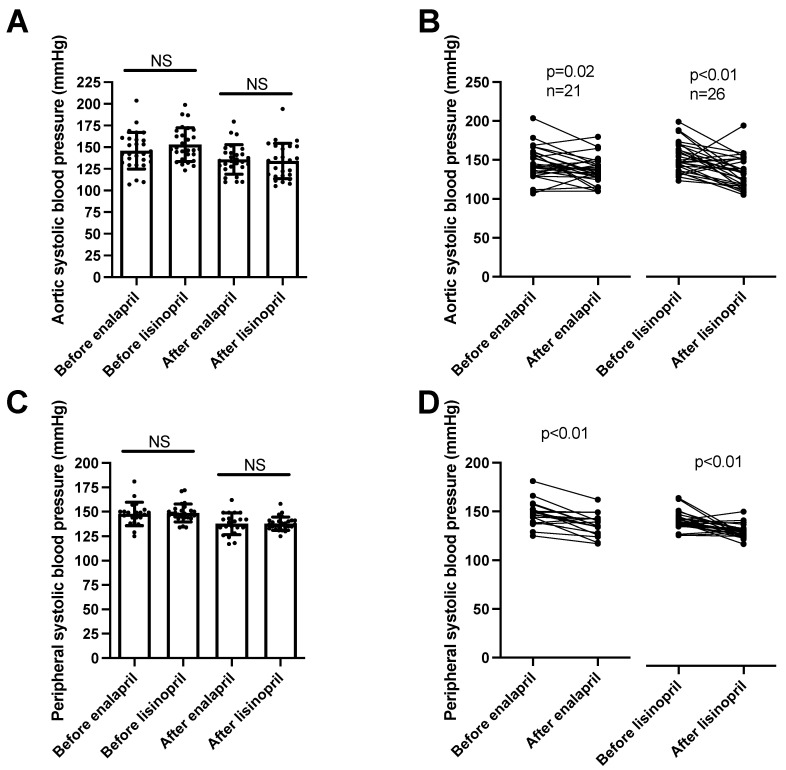
Same anti-hypertensive effects of enalapril and lisinopril on systolic blood pressure in hypertensive patients. Newly identified hypertensive patients were enrolled in the study at the outpatient facility of the Department of Neurology. Systolic blood pressure values were determined using 24 h ambulatory blood pressure monitoring (peripheral blood pressure) and arteriography (aortic blood pressure). Each symbol represents an individual patient. Bars represent the mean and SD for aortic (panel (**A**)) and peripheral (panel (**C**)) systolic blood pressure values. No significant differences were found among the groups (indicated by NS) by Kruskal–Wallis tests. Lines connect values before and after enalapril or lisinopril treatment in the case of aortic (panel (**B**)) and peripheral (panel (**D**)) systolic blood pressure values. Significant differences between values determined before and after the initiation of ACEi medication are labeled by the *p* values. Statistical differences were calculated by the Wilcoxon test.

Aortic systolic blood pressure (determined by the arteriography) levels were similar among the groups (enalapril before: 148 ± 3, after: 138 ± 2 mmHg, mean ± SEM, *n* = 21; lisinopril before: 149 ± 2, after: 138 ± 1 mmHg, mean ± SEM, *n* = 26; Figure 3A) and were similarly reduced by both ACEi drugs (Figure 3B). Peripheral systolic blood pressure values were measured using 24 h ambulatory blood pressure monitoring (ABPM). There were no significant differences among the patient groups (enalapril before: 146 ± 4, after: 136 ± 3 mmHg, mean ± SEM, *n* = 28; lisinopril before: 153 ± 4, after: 134 ± 4 mmHg, mean ± SEM, *n* = 28; Figure 3C), while both ACEi treatments uniformly resulted in significant reductions in systolic blood pressure values (Figure 3D).

**Figure 4:**
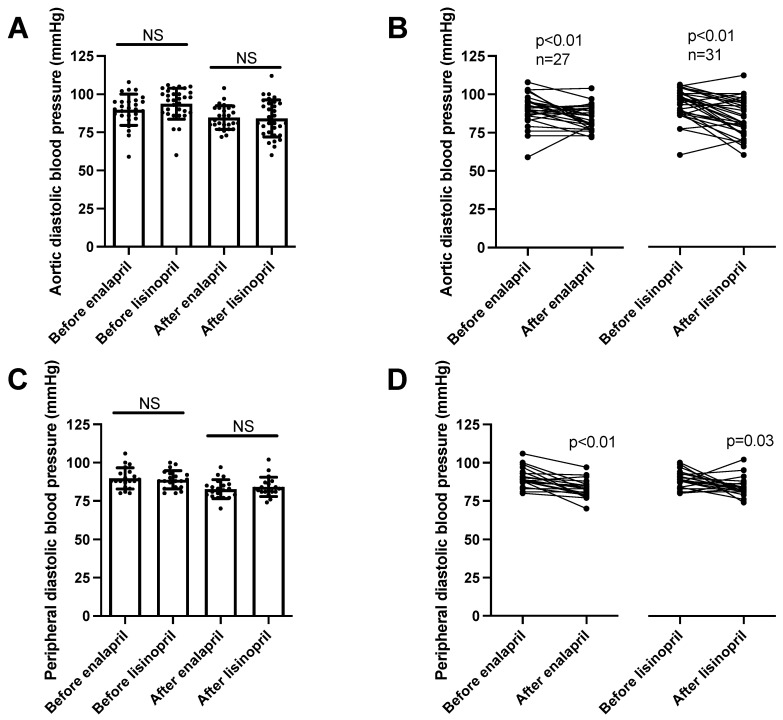
Same anti-hypertensive effects of enalapril and lisinopril on systolic blood pressure in hypertensive patients. Newly identified hypertensive patients were enrolled in the study at the outpatient facility of the Department of Neurology. Diastolic blood pressure values were determined using 24 h ambulatory blood pressure monitoring (peripheral blood pressure) and arteriography (aortic blood pressure). Each symbol represents an individual patient. Bars represent the mean and SD for aortic (panel (**A**)) and peripheral (panel (**C**)) diastolic blood pressure values. No significant differences were found among the groups (indicated by NS) by Kruskal–Wallis tests. Lines connect values before and after enalapril or lisinopril treatment in the case of aortic (panel (**B**)) and peripheral (panel (**D**)) diastolic blood pressure values. Significant differences between values determined before and after the initiation of ACEi medication are labeled by the *p* values. Statistical differences were calculated by the Wilcoxon test.

Diastolic pressure values mimicked the systolic ones. There were no differences among the patient groups in aortic (enalapril before: 90 ± 2, after: 85 ± 1 mmHg, mean ± SEM, *n* = 27; lisinopril before: 94 ± 2, after: 84 ± 2 mmHg, mean ± SEM, *n* = 27; Figure 4A) or peripheral diastolic blood pressure values (enalapril before: 90 ± 2, after: 83 ± 1 mmHg, mean ± SEM, *n* = 20; lisinopril before: 89 ± 1, after: 84 ± 1 mmHg, mean ± SEM, *n* = 20; Figure 4C). Both ACE inhibitory drugs reduced the aortic (Figure 4B) and peripheral (Figure 4D) blood pressure values.

**Figure 5:**
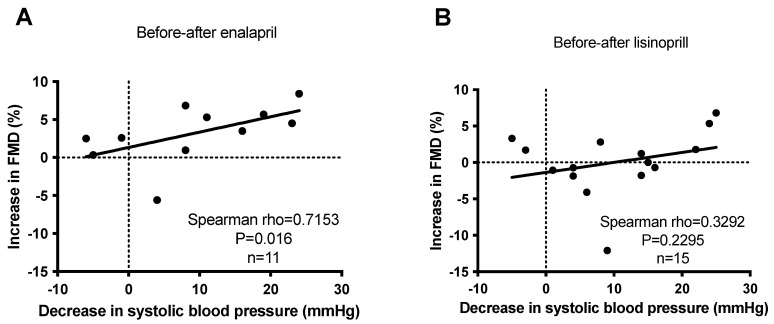
No correlation between anti-hypertensive effects and endothelial function improvement (FMD) upon short-term ACEi treatment in hypertensive patients. Newly identified hypertensive patients were enrolled in the study at the outpatient facility of the Department of Neurology. Increases in flow-mediated dilation (FMD) were plotted as the function of the decrease in systolic blood pressure before and after enalapril (panel (**A**)) and lisinopril (panel (**B**)) treatments. Each symbol represents an individual patient. The correlation between the parameters was tested by Spearman’s test. Correlations were characterized statistically by the rho and *p* values. Correlation is considered to be significant when *p* < 0.05.

Finally, the reduction in systolic blood pressure values was correlated with the improvement (increase) in flow-mediated dilation values in the case of enalapril. A strong (*rho* = 0.72) and significant (*p* = 0.016) correlation was established between the decrease in systolic blood pressure and increase in flow-mediated dilation when treated with enalapril (Figure 5A). In contrast, no significant correlation (*p* = 0.23) was observed in patients treated with lisinopril (Figure 5B).

## 4. Discussion

ACEi drugs represent one of the most frequently prescribed medications, being the primarily chosen drugs in chronic diseases affecting large populations, like hypertension [1,2] and heart failure [3,4]. ACEi prescriptions increase from 11.4% (40–59 years) to 21.3% (60–79 years) upon aging in the population [24]. In spite of the clinical success of ACE inhibition, the mechanism of action is still not fully understood [25,26]. An important example of this is the site of action of ACEi medical drugs. Obviously, the basis of the beneficial effects of ACEi drugs is the inhibition of ACE activity. However, recently, it was shown that physiological ACE activity is regulated by endogenous inhibitors in the circulation (blood) [11], as well as in cardiac and lung tissues [27]. Serum albumin was identified as a major endogenous inhibitor in the human blood, completely inhibiting circulating ACE activity under physiological conditions [13]. This suggested that ACEi drugs cannot act on the circulating enzyme, since ACE activity is negligible under physiological serum albumin concentrations. Indeed, the serum albumin sensitivity of the vascular tissue-bound form of ACE was lower, suggesting that tissular ACE may be the target of ACEi drugs [13]. In line with these, we hypothesized that ACEi drugs with different lipophilicities and affinities for carrier proteins have different physiological effects. To investigate this hypothesis, we tested the clinical effects of the most hydrophilic ACEi drug lisinopril (with low binding to carrier proteins) and contrasted it to the lipophilic ACEi drug enalapril.

A strength of this study was that we recruited patients upon a diagnosis of hypertension. Basal clinical parameters were recorded upon the initiation of ACEi treatment and after the administration of the drugs for at least 30 days. Accordingly, we prospectively recruited our patient population, in which the effects of the ACEi drugs could be tested before and after treatment in the same individuals. This setup is particularly useful in addressing delicate processes, such as vascular responsiveness. Endothelial function was tested by measuring the flow-mediated dilation in the brachial artery (FMD) [28], while arterial stiffness was tested using arteriography [29]. The major finding of this study is that, while enalapril dramatically improved endothelial function, the hydrophilic lisinopril was without effects. It was reported that blood pressure control correlates with an improvement in endothelial function [30]. Some of our results confirmed this observation by showing a strong correlation between the blood-pressure-lowering effect of enalapril and a parallel improvement in endothelial function, as measured by FMD [31]. However, we also found that the blood-pressure-lowering effect is independent of an improvement in FMD in the case of the hydrophilic ACEi inhibitor lisinopril. These findings suggest that an improvement in endothelial function is not uniquely mediated by the blood-pressure-lowering effects of ACE inhibition. There appears to be selective targeting of the tissular ACE population linked to endothelial dysfunction by the lipophilic enalapril.

This is in accordance with findings correlating vascular ACE inhibition (measured by changes in angiotensin peptide levels) [32] or vascular ACE expression (determined by genetic polymorphism) [33] with endothelial function. Moreover, the inhibition of ACE (and type 1 angiotensin 2 receptor) is associated with better endothelial function than that in patients treated with calcium channels or beta receptor blockers [31,34]. Others have also reported an improvement in endothelial function in patients treated with enalapril [35]. They hypothesized that the over-activation of vascular ACE may contribute to the increased production of superoxide, which neutralizes the endothelial NO, antagonizing endothelium-dependent dilations [35]. ACE may also interfere with inflammation [36], affecting endothelial functions (probably by regulating reactive oxygen radical formation). Finally, it should be noted that, quinapril, an exceptionally lipophilic ACEi, had a higher efficacy than enalapril in improving endothelial function [36,37]. Taken together, the reversal of endothelial function by ACEi drugs may depend on the tissue affinity (lipophilicity).

Our results suggested that short-term ACEi treatment does not affect the vascular structure, as there was no difference in carotid artery intima-media thickness before and after the ACEi drug treatment. Similar findings were noted for arterial stiffness, as the ACEi treatment was without effects on the augmentation index and pulse wave velocity determined using arteriography. In general, it can be concluded that the initiation of ACE inhibitor therapy resulted in highly significant changes in functional parameters (such as blood pressure or endothelial function), while it did not affect structural parameters significantly at this time point. One would speculate that functional improvements would be followed by structural remodeling during treatment in the long term when all drugs are up titrated [5].

We noted a significant blood-pressure-lowering effect of both enalapril and lisinopril. Both ACEi drug treatments resulted in a robust decrease in both peripheral (24 h ambulatory blood pressure monitoring) and aortic blood pressure (measured using arteriography). Both systolic and diastolic blood pressure values were decreased by both ACEi drugs, resulting in a similar decline in both the aortic and peripheral blood pressure values. Nonetheless, there was some difference in the decrease in pulse pressure (difference in systolic and diastolic blood pressure values). While both ACEi drugs reduced aortic pulse pressure, only enalapril was able to significantly reduce peripheral pulse pressure. These apparent differences between enalapril and lisinopril may also reflect the different levels of tissular ACE inhibition, which may be attributed to the more lipophilic nature of enalapril.

Our results showing a robust anti-hypertensive effect for both enalapril and lisinopril are in accordance with previous reports, suggesting a uniform class effect for ACEi drugs [5,10,30,38]. This is important in light of the differences noted in vascular responsiveness. Both ACEi drugs had robust and similar anti-hypertensive effects. The anti-hypertensive effects of ACE inhibition were attributed to various mechanisms, including improved endothelial function, increased bradykinin levels, suppressed vascular smooth muscle contraction, and decreased volume overload, mediated by facilitated natriuresis [39]. Our results showed that the anti-hypertensive effects of ACEi drugs are not primarily mediated by improving endothelial function or arterial stiffness. This suggests that the modulation of kidney function (increased natriuresis and consequent reduction in volume overload) is the primary mechanism behind the anti-hypertensive effects of ACEi.

ACEIs can easily cause side effects such as dry cough, angioedema, rash, changes in taste, increased blood potassium, and decreased kidney function [5]. Some may note that the results of this paper do not seem to mention these phenomena. These side effects most probably did not have time to develop, since the study represents the initial examination (freshly identified untreated hypertensives) and their first visit after the prescription of low-dose ACE inhibitors (randomly, enalapril and lisinopril). It is most likely that these side effects would develop during the up-titration and long-term use of ACE inhibitory drugs in these patients.

In summary, here we showed that, while enalapril and lisinopril have similar anti-hypertensive effects, they have different effects on endothelial function. In particular, the lipophilic enalapril improved endothelial function, while the hydrophilic lisinopril was without effects. These findings suggest that the blood-pressure-lowering class effect of ACEi drugs is mediated by improvements in kidney function and decreased volume overload. On the other hand, ACEi drugs do not have a class effect on improving endothelial functions (either mediated by reduced angiotensin 2 levels or by increased bradykinin levels).

A strength of this report over previous investigations is that we verified the ACEi treatment efficacy using objective biochemical enzyme activity measurements. Another advantage is that we performed a prospective study to identify differences among ACEi drugs with different pharmacological properties. Note that the study was randomized in a way that patients were recruited consecutively (odd numbers selected for one drug even numbers for the other), although patients and doctors were not blinded. This randomization is very important, since, otherwise, one may have a potential problem of confounding by indication. When a drug appears to be associated with an outcome, the outcome may, in fact, be caused by the indication for which the drug was used, or some factor associated with the indication. This can only be avoided by randomization.

Having said that, all clinical studies have limitations. Here, we investigated a patient population in a single-center study, involving a homogenous (Caucasian) population. Accordingly, our results cannot be extrapolated to all human populations. Moreover, the study consisted of patients first identified with hypertension and patients in the early stages of their anti-hypertensive treatment. The relevance of this is that the treatment regimen started with a low dose of ACE inhibitors, and there was no up-titration in the upcoming visits (since the study represents the initial visit and the first upcoming visit). Note that initial low-dose ACE inhibitors usually provide the full anti-hypertensive effects, while the up-titration of the drug is important to improve long-term efficacy and surrogate endpoints, such as in heart failure. We encountered a significant dropout rate in our study, resulting in a relatively small patient population in certain instances, thereby impacting its statistical power. Future clinical investigations could address these limitations and build on our methodological framework.

## 5. Conclusions

These data suggest that hydrophilicity defines the site of action of ACE inhibitors. Lipophilic inhibitors improve endothelial function, while hydrophilic ones do not. The blood pressure-lowering effect of ACE inhibitors is independent of hydrophilicity. The blood pressure lowering is independent of improvement in endothelial function.

## Figures and Tables

**Table 1:** Medical characteristics of study populations.

Medical Drug	Enalapril		Lisinopril	
Visit	First Visit	Follow Up	First Visit	Follow Up
Involved patients (*n*)	43	28	44	30
Age (years)	45.7 ± 11.1	N/A	45.1 ± 11.3	N/A
BMI (kg/m^2^)	28.9 ± 5.6	N/A	28.3 ± 3.8	N/A
Dose (mg/day)	0	5.5 ± 2.3	0	8.3 ± 3.0
Na^+^ (mM)	140.6 ± 2.0	140.0 ± 2.5	140.3 ± 2.1	139.7 ± 2.5
K^+^ (mM)	4.3 ± 0.3	4.3 ± 0.3	4.2 ± 0.3	4.4 ± 0.4
Glucose (mM)	5.5 ± 1.0	5.2 ± 0.6	5.2 ± 1.3	5.2 ± 1.3
HbA1C (%)	5.3 ± 0.5	5.2 ± 0.3	5.4 ± 0.8	5.4 ± 0.8
Urea (mmol/L)	5.2 ± 1.4	5.4 ± 1.4	4.8 ± 1.2	5.0 ± 1.4
Creatinine (mg/dL)	75 ± 15	74 ± 15	78 ± 17	79 ± 16
Trigliceride (mmol/L)	1.5 ± 0.9	1.6 ± 1.5	2.1 ± 3.0	1.5 ± 0.8
Cholesterol (mmol/L)	5.1 ± 0.9	5.1 ± 0.9	5.2 ± 1.1	5.3 ± 1.1
GOT (IU/L)	25.2 ± 11.8	26.4 ± 9.6	22.8 ± 7.5	23.1 ± 7.2
Systolic blood pressure (active period, mmHg, SD)	147 ± 11	138 ± 11	148 ± 11	139 ± 8
Diastolic blood pressure (active period, mmHg, SD)	90 ± 7	83 ± 7	90 ± 9	84 ± 6
Systolic blood pressure (night period, mmHg, SD)	128 ± 13	125 ± 14	132 ± 23	118 ± 27
Diastolic blood pressure (night period, mmHg, SD)	76 ± 8	75 ± 8	80 ± 10	70 ± 17
FMD (%, SD)	7.4 ± 3.1	8.8 ± 3.4	8.7 ± 4.2	7.8 ± 2.4
IMT (mm, SD)	0.6 ± 0.1	0.6 ± 0.1	0.6 ± 0.1	0.6 ± 0.1

## Data Availability

Data are contained within the article.

## Abbreviations

ABPM	Ambulatory blood pressure monitoring
ACE	Angiotensin-converting enzyme
ACEi	Angiotensin-converting enzyme inhibitors
AIx	Augmentation Index
FMD	Flow-mediated dilation
PWV	Pulse Wave Velocity
